# Study on Coupling Correlation of Factors Affecting Mechanical Properties of High-Damping Rubber Plate

**DOI:** 10.3390/polym17050593

**Published:** 2025-02-23

**Authors:** Tianbo Peng, Feng Li, Chiyuan Ma

**Affiliations:** 1 State Key Laboratory of Disaster Reduction in Civil Engineering, Tongji University, Shanghai 200092, China; ptb@tongji.edu.cn (T.P.); 2232434@tongji.edu.cn (C.M.); 2 College of Civil Engineering, Tongji University, Shanghai 200092, China

**Keywords:** high-damping rubber, temperature, shear strain rate, shear strain amplitude, coupling correlation

## Abstract

Factors affecting the mechanical properties of the high-damping rubber plate mainly include temperature, shear strain rate, shear strain amplitude, and compressive stress. However, the existing studies focused on one factor and have not conducted detailed research on the coupling relationships between various factors. The influence on the mechanical properties of the high-damping rubber plate of one factor will change with the change in other factors. To study the coupling correlation, a series of horizontal cyclic loading tests were designed and carried out. Due to the simultaneous hyperelastic and viscoelastic properties of high-damping rubber, the hysteresis curve of the high-damping rubber plate is decomposed into a hyperelastic part and a damping part. Equations of the two parts are deduced by a theoretical method, and all the coefficients of the two parts are obtained by fitting the test results. Then, the variation coefficient is used to study the influence degree of each factor on these coefficients. Finally, the correlation variation coefficient is defined as the variation coefficient of variation coefficients and is used to quantify the coupling correlation between the factors. This study is of great significance in exploring the coupling correlation between the factors affecting the mechanical properties of the high-damping rubber plate.

## 1. Introduction

Benefiting from the simple configuration, easy fabrication, and a certain capacity of energy consumption, the high-damping rubber (HDR) bearing won plentiful applications in civil engineering seismic isolation design at home and abroad. The HDR bearing is made of laminated HDR plates and steel plates, and its deformation ability and energy consumption capacity are mainly provided by HDR plates. As a viscoelastic material, the mechanical properties of HDR plates are very susceptible to multiple factors. Therefore, a lot of research on the issue has been conducted.

The conclusions of previous studies on the effect of temperature are generally consistent. It was found that the horizontal equivalent stiffness and the equivalent damping ratio decrease with increasing temperature. The influence of temperature on the mechanical properties of HDR is decisive [1,2,3,4,5,6,7]. Chen et al. [8] studied the effect of three different temperatures, namely 0 °C, 23 °C, and 40 °C, on the shear performance of HDR bearings. In the test conducted by Shen et al. [9], the temperature range is from −20 °C to 40 °C, while in the test conducted by Gao et al. [10], the temperature range is from −40 °C to 40 °C. In [11], simple shear, single-step, and multi-step stress relaxation tests were conducted on HDR bearings at different temperatures of −30 °C, −10 °C, and 23 °C, respectively. Xu et al. [12] conducted tests at −13.9 °C, 0.2 °C, 10.8 °C, 15.2 °C, 24.6 °C, and 31.5 °C. Scholars also studied the coupling relationship between HDR bearings and structures, and discussed the influence of self-generated heat on the mechanical properties of HDR bearings during energy dissipation [1,13,14,15].

For the effect of loading frequency (or loading rate), the researchers [11,12,16,17,18,19,20,21,22,23,24,25,26,27,28] revealed that the horizontal equivalent stiffness of the bearing increases with the increasing loading frequency. However, Refs. [8,9,29] found that the loading frequency has little effect on the horizontal equivalent stiffness of the bearing. According to the researchers [8,9,17,18], the equivalent damping ratio of the bearing increases with the increasing loading frequency, and [16,19,29] found that the loading frequency has little effect on the equivalent damping ratio.

There are some divergences in the conclusions of previous studies regarding the effect of shear strain amplitude mainly due to the differences in the compositions and processing technology of HDR material. According to the research [8,12,16,17,20,21,23,26,30,31,32,33], the horizontal equivalent stiffness decreases with the increasing shear strain. According to the research [9,18,29,34,35], the horizontal equivalent stiffness decreases first and then increases with the increasing shear strain. The decrease in the horizontal equivalent stiffness in the former segment is mainly because the core compression area shrinks with the increasing shear strain, while the rubber outside the core compression area provides a less horizontal restoring force. The increase in the horizontal equivalent stiffness in the latter segment is mainly due to the large strain hardening phenomenon of the HDR plates. However, according to the research of Gao et al. [10], the horizontal equivalent stiffness decreases with the increasing shear strain. The equivalent damping ratio is defined as the ratio of the hysteretic energy to the elastic energy storage. Most scholars found that the equivalent damping ratio of the bearing decreases with the increasing shear strain [8,9,10,16,18,29,30,34,35]. However, Chen et al. [17] and Li et al. [23] found that the equivalent damping ratio of the bearing will increase slightly with the increasing shear strain. Oliveto et al. [36] also studied the response of high-damping rubber bearings under low, medium, and high strain levels during bidirectional loading.

There are also some divergences in the effect of compressive stress. Considering the influence of compressive stress is crucial for a proper understanding of the behavior of HDR under cyclic shear. In practical situations, HDR bearings bear permanent vertical loads that may change during earthquakes and undergo shear deformation simultaneously due to structural service movements or ground movements caused by earthquakes [37]. Most scholars [8,9,10,17,25,29,30,38] found that increasing the compressive stress will compact the HDR plates, thus increasing the horizontal equivalent stiffness of the bearing. According to the research [18,34,35], the horizontal equivalent stiffness of the bearing decreases with increasing compressive stress. In terms of the equivalent damping ratio, most scholars [8,9,10,17,18,35] found that the increased compressive stress will increase the internal friction of the HDR plates, which will increase the equivalent damping ratio. However, Zhuang et al. [30] and Jiang et al. [34] found that the equivalent damping ratio decreases with the increase in compressive stress.

Previous scholars focused on the influence of one single factor, such as temperature, loading frequency, shear strain, and compressive stress, on the shear performance of HDR bearings, and gave some conclusions. However, few studies have been conducted in the study of the coupling correlation of the factors.

To study the coupling correlation of the factors, six groups of cyclic loading tests for HDR plates are designed. The theoretical equations are established by decomposing the hysteresis curve of the HDR plate into a hyperelastic part and a damping part first. Then, the coefficients of the equations are obtained by fitting the test results. The relationships between the coefficients and the factors are obtained by the definition of the variation coefficient. The coupling correlations of factors are studied quantitatively by the definition of correlation variation coefficient. This study provides a theoretical basis for accurately describing the hysteresis behavior of HDR in scientific research and engineering applications. Although the HDR used in this study is based on only one rubber formulation, it still has high research reference value for HDR of other rubber formulations. The novelty of this study lies in the in-depth investigation of the coupling relationship between various factors by decomposing the hysteresis curve and defining the correlation variation coefficient.

## 2. Materials and Methods

### 2.1. Test Specimen

**Figure 1:**
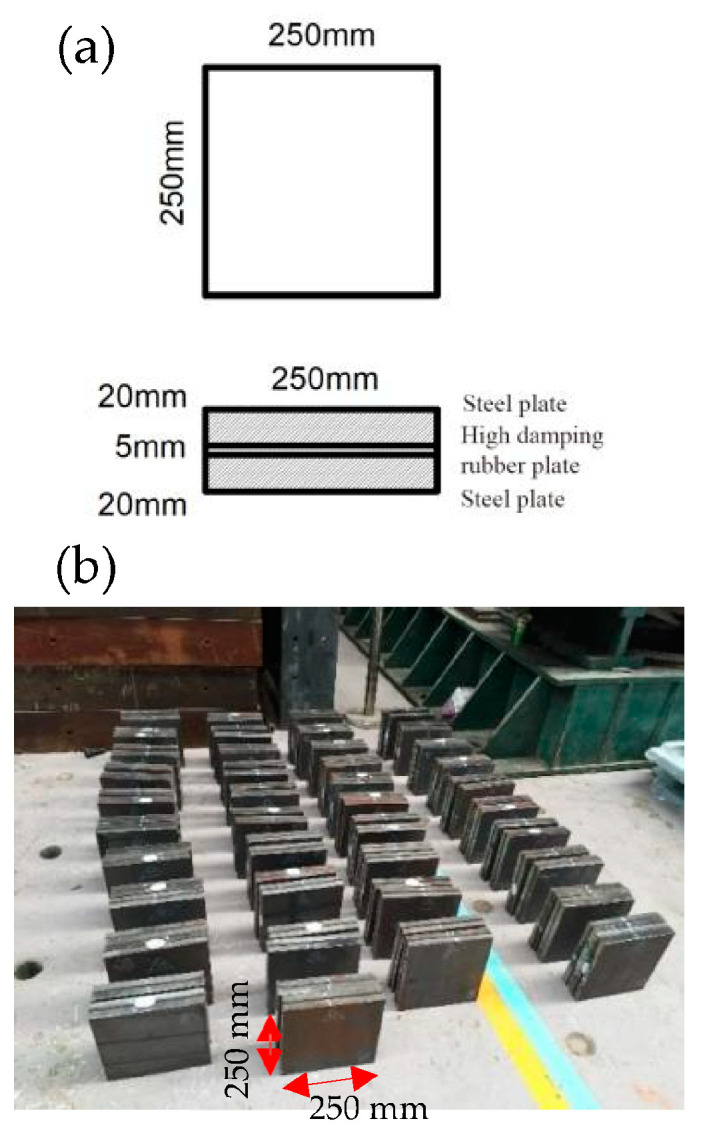
Test specimens. (**a**) Dimensional drawing of test specimen; (**b**) physical photos of test specimens.

**Figure 2:**
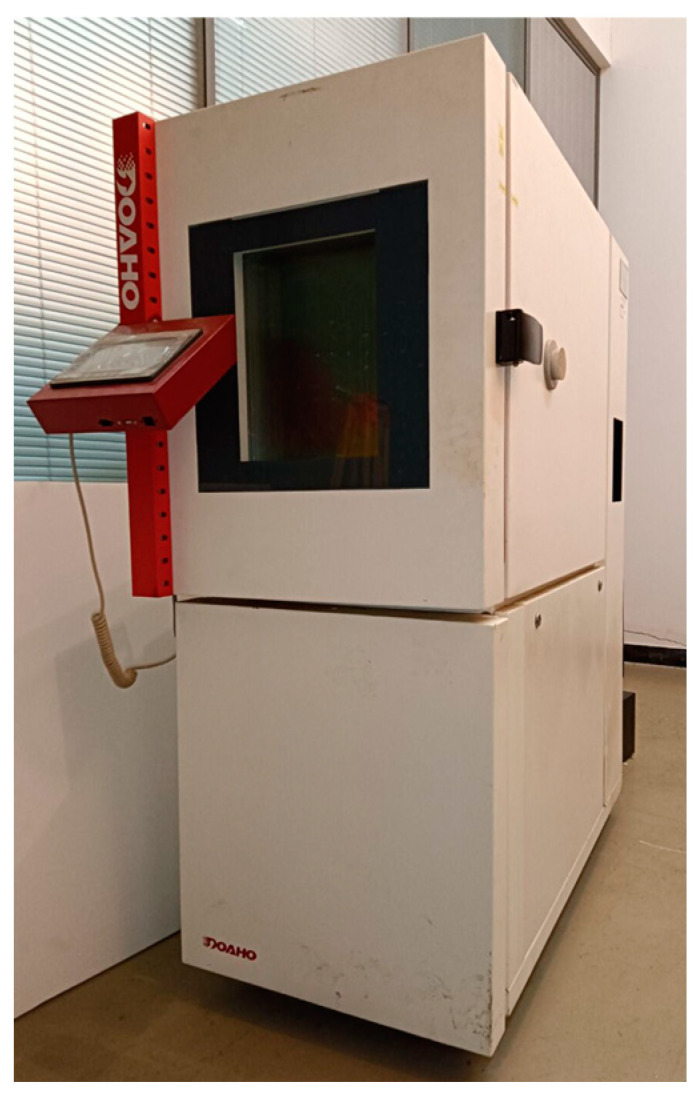
Temperature control box.

The test specimen is composed of two steel plates and an HDR plate. The three plates are stuck together by hot pressing, as shown in Figure 1. The plane size is 250 mm × 250 mm. The thickness of the steel plate is 20 mm, that of the HDR plate is 5 mm, and the total thickness of the specimen is 45 mm. The temperature control box manufactured by Shanghai DOAHO Test Equipment Factory (Shanghai, China) is used to adjust the temperature of the specimen, as shown in Figure 2.

### 2.2. Test Device

**Figure 3:**
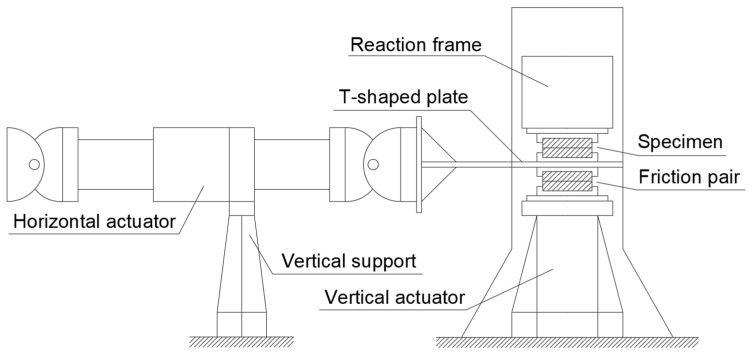
The test device.

The test device is shown in Figure 3. The vertical actuator is used to apply the vertical load. The horizontal cyclic load is applied by an electro-hydraulic servo actuator.

### 2.3. Test Cases

To ascertain the relationship between the coefficients and the factors, six groups of horizontal cyclic loading tests are designed, as listed in Table 1. The factor values of all the cases have been calculated according to the potentialities of HDR plates in the event of an earthquake, as well as the loading capacity of the test devices.

**Figure 4:**
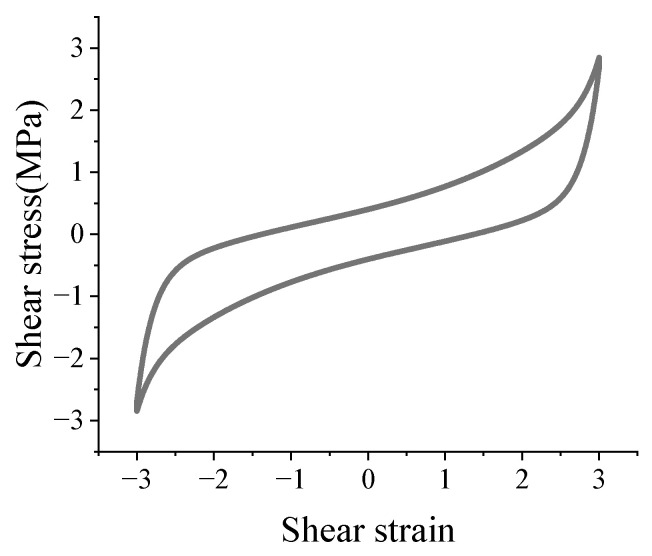
Typical hysteresis curve of an HDR plate.

The typical hysteresis curve of an HDR plate obtained from the test is shown in Figure 4.

### 2.4. Theoretical Equations

**Figure 5:**
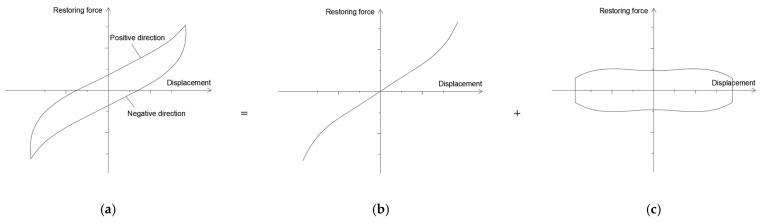
Decomposition of a typical hysteresis curve. (**a**) Hysteresis curve; (**b**) hyperelastic part; and (**c**) damping part.

The typical hysteresis curve of an HDR plate is shown in Figure 5a. It can be seen that the hysteresis curve is central symmetric and includes two loading segments in the positive direction and the negative direction. To accurately study the hysteresis behavior of the HDR plate, the hysteresis curve is decomposed into a hyperelastic part and a damping part. The hyperelastic part is obtained by averaging the two loading segments, and the damping part is obtained by subtracting the hyperelastic part from the hysteresis curve, as shown in Figure 5b,c.

#### 2.4.1. Hyperelastic Part

**Figure 6:**
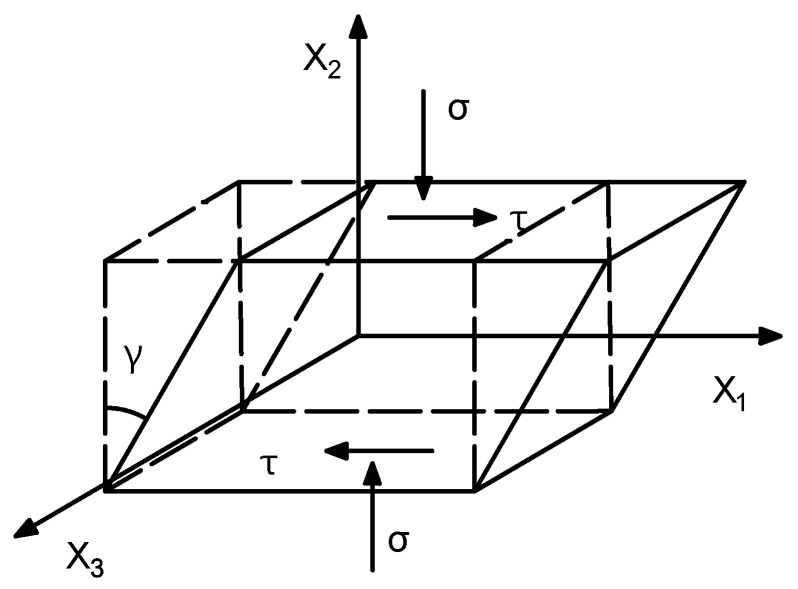
Compression–shear deformation of an HDR element.

The hyperelastic part characterizes the nonlinear stiffness of HDR. In this section, the equation of the hyperelastic part of an HDR element under compression–shear loading is derived. The dashed line indicates the undeformed state of the HDR element, and the solid line indicates the state of the HDR element after the compression–shear deformation, as shown in Figure 6. The origin of the coordinate system is located in the center of the HDR element.

The coordinates after the deformation are as follows:

(1) $$\left\{\begin{array}{l} x_{1}=(1-\upsilon \varepsilon )X_{1}+\gamma X_{2}\\ x_{2}=(1+\varepsilon )X_{2}\\ x_{3}=(1-\upsilon \varepsilon )X_{3} \end{array}\right.$$

where *γ* is the shear strain, *ε* is the positive strain, *υ* is the Poisson’s ratio, *x_i_* (*i* = 1, 2, 3) is the current configuration coordinate, and *X_I_*(*I* = 1, 2, 3) is the reference configuration coordinate.

The deformation gradient tensor *F* is as follows:

(2) $$F=\frac{\partial (x_{1},x_{2},x_{3})}{\partial (X_{1},X_{2},X_{3})}=\left[\begin{array}{ccc} \frac{\partial x_{1}}{\partial X_{1}} & \frac{\partial x_{1}}{\partial X_{2}} & \frac{\partial x_{1}}{\partial X_{3}}\\ \frac{\partial x_{2}}{\partial X_{1}} & \frac{\partial x_{2}}{\partial X_{2}} & \frac{\partial x_{2}}{\partial X_{3}}\\ \frac{\partial x_{3}}{\partial X_{1}} & \frac{\partial x_{3}}{\partial X_{2}} & \frac{\partial x_{3}}{\partial X_{3}} \end{array}\right]=\left[\begin{array}{ccc} 1-\upsilon \varepsilon & \gamma & 0\\ 0 & 1+\varepsilon & 0\\ 0 & 0 & 1-\upsilon \varepsilon \end{array}\right].$$

The left Cauchy–Green deformation tensor *B* is as follows:

(3) $$B=F\cdot F^{T}=\left[\begin{array}{ccc} {\left(1-\upsilon \varepsilon \right)}^{2}+\gamma^{2} & (1+\varepsilon )\gamma & 0\\ (1+\varepsilon )\gamma & {\left(1+\varepsilon \right)}^{2} & 0\\ 0 & 0 & {\left(1-\upsilon \varepsilon \right)}^{2} \end{array}\right].$$

The strain invariants *I*_1_, *I*_2_, and *I*_3_ are represented by the left Cauchy–Green deformation tensor *B* so that the strain energy function does not depend on the coordinate system and direction.

(4) $$\left\{\begin{array}{l} I_{1}=trB\\ I_{2}=\frac{1}{2}[{\left(trB\right)}^{2}-(trB^{2})]\\ I_{3}=\det B \end{array}\right.$$

where *tr* is the trace of the matrix and *det* is the determinant of the matrix.

The strain invariants *I*_1_, *I*_2_, and *I*_3_ can be obtained from Equation (4).

(5) $$\left\{\begin{array}{l} I_{1}=\gamma^{2}+(1+\varepsilon {)}^{2}+2(1-\upsilon \varepsilon {)}^{2}\\ I_{2}=\gamma^{2}(1-\upsilon \varepsilon {)}^{2}+(1-\upsilon \varepsilon {)}^{4}+2(1-\upsilon \varepsilon {)}^{2}(1+\varepsilon {)}^{2}\\ I_{3}=(1-\upsilon \varepsilon {)}^{4}(1+\varepsilon {)}^{2} \end{array}\right.$$

In general, *ε* is the higher-order small quantity of *γ*^2^. According to the incompressibility of rubber, *υ* = 0.5, thus Equation (5) can be simplified as follows:

(6) $$\left\{\begin{array}{l} I_{1}\approx 3+\gamma^{2}\\ I_{2}\approx 3+\gamma^{2}\\ I_{3}\approx 1 \end{array}\right..$$

The mechanical properties of HDR are often expressed by the strain energy density function. Commonly used strain energy density functions include the Mooney model [39], Mooney–Rivlin model [40], neo-Hookean model [41], James model [42], Green model [43], Yeoh model [44], etc. Based on these models, Wei [45] proposed a strain energy density function of HDR with high precision from small strain to large strain. The equation of Wei’s model is as follows:

(7) $$W=C_{10}(I_{1}-3)+C_{20}{\left(I_{1}-3\right)}^{\frac{3}{2}}+C_{30}{\left(I_{1}-3\right)}^{2}+C_{40}{\left(I_{1}-3\right)}^{\frac{5}{2}}.$$

In this paper, Equation (7) is used as the strain energy function. Substitute Equation (6) into Equation (7), and differentiate the shear strain, and thus the shear stress–strain relationship of the hyperelastic part can be obtained:

(8) $$\tau =C_{1}^{\prime }\gamma +C_{2}^{\prime }\gamma^{2}\mathrm{sgn}(\gamma )+C_{3}^{\prime }\gamma^{3}+C_{4}^{\prime }\gamma^{4}\mathrm{sgn}(\gamma ).$$

For ease of introduction, the shear strain ratio *x* is defined as the ratio of shear strain *γ* to shear strain amplitude *γ*_p_; that is, *x* = *γ*/*γ*_p_. Substitute *x* into Equation (8), and thus the equation of the hyperelastic part can be transformed as follows:

(9) $$\tau =C_{1}x+C_{2}x^{2}\mathrm{sgn}(x)+C_{3}x^{3}+C_{4}x^{4}\mathrm{sgn}(x)$$

where *C_i_* (*i* = 1, 2, 3, 4) is the hyperelastic coefficient fitted according to the test results, and sgn() is the sign function. The size of *C*_1_ is related to the initial modulus, which controls the initial linear segment of the hyperelastic part. *C*_2_, *C*_3_, and *C*_4_ control the shape of the nonlinear strain-hardening segment after the initial linear segment.

#### 2.4.2. Damping Part

The damping part represents the energy dissipation capacity of HDR, and the shear stress–strain relationship of the damping part is generally expressed by Equation (10) [46].

(10) $$\tau =C_{5}^{\prime }\mathrm{sgn}(v){\left|v\right|}^{a}$$

Judging from the damping part, the curve is not only related to the shear strain rate, but also to the shear strain. According to the test results, the damping part of HDR can be expressed by Equation (11).

(11) $$\tau =[C_{5}+C_{6}x^{2}+C_{7}(e^{x}+e^{-x}-2)]\mathrm{sgn}(v)v^{0.24}$$

where *C_i_* (*i* = 5, 6, 7) is the damping hysteresis coefficient fitted according to the test results; *ν* is the shear strain rate and sgn() is the sign function. *C*_5_ is related to shear stress at *γ* = 0; that is, characteristic stress. *C*_6_ and *C*_7_ control the shape of the nonlinear strain-hardening segment.

## 3. Results and Discussion

### 3.1. Variation in the Coefficients with Different Factors

A set of coefficients *C_i_* (*i* = 1~7) can be determined by fitting the hyperelastic part by Equation (9) and the damping part by Equation (11). Therefore, seventy-two sets of coefficients are obtained as shown in Table A1, Table A2, Table A3, Table A4, Table A5 and Table A6.

The coefficients (*C*_1_, *C*_2_, *C*_3_, and *C*_4_) of the hyperelastic part are negatively correlated with temperature, positively correlated with shear strain rate and shear strain amplitude, and fluctuate slightly with compressive stress. The slope of the initial linear segment increases with decreasing temperature, increasing shear strain rate, and shear strain amplitude, while it does not change with compressive stress. The coefficients (*C*_5_, *C*_6_, and *C*_7_) of the damping part are negatively correlated with temperature and positively correlated with shear strain rate, shear strain amplitude, and compressive stress. From the coefficient *C*_5_, it can be seen that the characteristic stress increases with decreasing temperature, increasing shear strain rate, shear strain amplitude, and compressive stress.

### 3.2. Variation Coefficient

In this study, the influence degree of a factor to a coefficient *C_i_* can be quantified by a variation coefficient, which is defined as Equations (12)~(15).

Suppose the correlation of factor *X* and factor *Y* is studied in Group *j* (*j* = 1~6). Where *X* has *m* values: *x*_1_, *x*_2_, …, *x_m_*, and *Y* has *n* values: *y*_1_, *y*_2_, …, and *y_n_*. Then, there are *m* × *n* coefficients *C_i_* (*i* = 1~7) in Group *j* (*j* = 1~6). For *X* = *x_k_* and *Y* = *y_l_*, the coefficient is symbolized by $C_{i,x_{k},y_{l}}$, as listed in Table 2.

$\sigma_{Ci,X,y_{l}}$ and $\mu_{Ci,X,y_{l}}$ are defined as the standard deviation and the average value of all the coefficients $C_{i,x_{k},y_{l}}$ (*k* = 1~*m*) when *Y* = *y_l_*. Then, $Cv_{i,X,y_{l}}$ is defined as the variation coefficient of factor *X* when *Y* = *y_l_*, which is equal to the absolute value of the ratio of $\sigma_{Ci,X,y_{l}}$ to $\mu_{Ci,X,y_{l}}$. It reflects the influence degree of factor *X* to the coefficient *C_i_* when *Y* = *y_l_*.

$\mu_{Cv_{i},X(Y)}$ is defined as the average value of variation coefficients, which reflects the average influence degree of factor *X* to the coefficient *C_i_* for various *Y*. The larger the average value of variation coefficients, the larger the influence of one factor on the coefficient *C_i_* when other factors are fixed.

(12) $$\mu_{C_{i},X,y_{l}}=\frac{1}{m}\sum_{k=1}^{m}C_{i,x_{k},y_{l}}$$

(13) $$\sigma_{C_{i},X,y_{i}}=\sqrt{\frac{1}{m}\sum_{k=1}^{m}{\left(C_{i,x_{k},y_{l}}-\mu_{C_{i},X,y_{l}}\right)}^{2}}$$

(14) $$Cv_{i,X,y_{l}}=\left|\frac{\sigma_{C_{i},X,y_{l}}}{\mu_{C_{i},X,y_{l}}}\right|$$

(15) $$\mu_{Cv_{i},X(Y)}=\frac{1}{n}\sum_{l=1}^{n}Cv_{i,X,y_{l}}.$$

Average values of variation coefficients can be calculated from the test results, as listed in Table 3.

It can be seen that the coefficients *C_i_* (*i* = 1~7) can be roughly divided into four types. For *C*_1_, the most influential factor is temperature and the average value of three groups (i.e., subgroup 1-1, 2-1, and 3-1) is 0.205. The second most influential factor is shear strain amplitude and the average value of three groups (i.e., subgroup 2-1, 4-2, and 6-1) is 0.162. The influence of the shear strain rate is relatively small and the average value of three groups (i.e., subgroup 1-2, 4-1, and 5-1) is 0.114. The influence of compressive stress is negligible and the average value of three groups (i.e., subgroup 3-2, 5-2, and 6-2) is 0.044.

Influence patterns of *C*_2_~*C*_4_ are the same. Shear strain amplitude has the greatest influence on the three coefficients and the average values of the three groups are 0.751, 0.711, and 0.697, respectively. The temperature has the second greatest influence on the three coefficients and the average values of the three groups are 0.477, 0.483, and 0.477, respectively. The shear strain rate has a relatively small influence on the three coefficients and the average values of the three groups are 0.191, 0.223, and 0.191, respectively. compressive stress has a negligible influence on the three coefficients and the average values of the three groups are 0.079, 0.074, and 0.079, respectively.

For *C*_5_, the most influential factor is temperature and the average value of the three groups is 0.436. The second most influential factor is shear strain amplitude and the average value of the three groups is 0.204. The influence of the shear strain rate is relatively small and the average value of the three groups is 0.129. The influence of compressive stress is negligible and the average value of the three groups is 0.090.

The influence patterns of *C*_6_ and *C*_7_ are also basically the same. The influences of temperature and shear strain amplitude on the two coefficients are both significant and the average values of the three groups are both larger than 0.467. The shear strain rate has a relatively small influence on the two coefficients and the average values of three groups of the two coefficients are both equal to 0.260. Compressive stress also has a certain influence on the two coefficients, and the average values of three groups of the two coefficients are 0.126 and 0.127, respectively.

Comparing the four factors, the influence of temperature on each coefficient is generally the greatest, especially for *C*_1_ and *C*_5_, which are related to the initial modulus and characteristic stress, respectively. Additionally, it is also important to the other five coefficients. Shear strain amplitude also has a considerable influence on each coefficient, especially on *C*_2_~*C*_4_, *C*_6,_ and *C*_7_, which control the shapes of nonlinear strain-hardening segments. However, it has less influence on *C*_1_ and *C*_5_ than temperature. The influence of shear strain rate on each coefficient is relatively slight, while the influence of compressive stress is almost negligible. The impact of these four factors on the mechanical properties of HDR is consistent with the conclusions of existing mainstream research.

### 3.3. Correlation Variation Coefficient

In this study, $\sigma_{Cvi,X(Y)}$ is defined as the standard deviation of variation coefficients $Cv_{i,X,y_{l}}$(*l* = 1~*n*). Additionally, $C_{w_{i},X(Y)}$ is then defined as the correlation variation coefficient of factor *Y* to *X*, which is equal to the absolute value of the ratio of $\sigma_{Cvi,X(Y)}$ to $\mu_{Cvi,X(Y)}$. The correlation variation coefficient can be considered as the variation coefficient of variation coefficients. It reflects the influence degree of factor *Y* to $Cv_{i,X,y_{l}}$, which is the variation coefficient of *X*. If $C_{w_{i},X(Y)}$ and $C_{w_{i},Y(X)}$, i.e., the correlation variation coefficient of *Y* to *X* and that of *X* to *Y*, is relatively small, *X* and *Y* are almost unrelated, otherwise they are correlated. The standard deviation of variation coefficients and the correlation variation coefficient are expressed as Equations (16) and (17).

(16) $$\sigma_{Cv_{i},X\left(Y\right)}=\sqrt{\frac{1}{n}\sum_{l=1}^{n}{\left(Cv_{i,X,y_{i}}-\mu_{Cv_{i},X\left(Y\right)}\right)}^{2}}$$

(17) $$Cw_{i,X(Y)}=\left|\frac{\sigma_{Cv_{i},X(Y)}}{\mu_{Cv_{i},X(Y)}}\right|$$

Correlation variation coefficients can also be calculated from the test results, as listed in Table 4.

As shown in Table 4, there is no case in which $C_{w_{i},X(Y)}$ and $C_{w_{i},Y(X)}$ are both are relatively small. That means there is no case in which two factors are completely unrelated.

The shear strain amplitude has the greatest influence on the influence pattern of other factors. The influence of shear strain amplitude on the influence pattern of temperature is shown in subgroup 2-1 and the average value of the correlation variation coefficient of the seven coefficients is 0.225. The influence of shear strain amplitude on the influence pattern of shear strain rate is shown in subgroup 4-1 and the average value of the correlation variation coefficient of the seven coefficients is 0.682. The influence of shear strain amplitude on the influence pattern of compressive stress is shown in subgroup 6-2 and the average value of the correlation variation coefficient of the seven coefficients is 0.316.

Temperature generally has the second greatest influence on the influence pattern of other factors. The influence of temperature on the influence pattern of shear strain rate is shown in subgroup 1-2 and the average value of the correlation variation coefficient of the seven coefficients is 0.223. The influence of temperature on the influence pattern of shear strain amplitude is shown in subgroup 2-2 and the average value of the correlation variation coefficient of the seven coefficients is 0.262. The influence of temperature on the influence pattern of compressive stress is shown in subgroup 3-2 and the average value of the correlation variation coefficient of the seven coefficients is 0.305.

Shear strain rate has a slight influence on the influence pattern of other factors. The influence of shear strain rate on the influence pattern of temperature is shown in subgroup 1-1 and the average value of the correlation variation coefficient of the seven coefficients is 0.042. The influence of shear strain rate on the influence pattern of shear strain amplitude is shown in subgroup 4-2 and the average value of the correlation variation coefficient of the seven coefficients is 0.248. The influence of shear strain rate on the influence pattern of compressive stress is shown in subgroup 5-2 and the average value of the correlation variation coefficient of the seven coefficients is 0.159.

The influence of compressive stress on the influence pattern of temperature is shown in subgroup 3-1 and the average value of correlation variation coefficients of the seven coefficients is 0.059. The influence of compressive stress on the influence pattern of shear strain rate is shown in subgroup 5-1 and the average value of correlation variation coefficients of the seven coefficients is 0.120. The influence of compressive stress on the influence pattern of shear strain amplitude is shown in subgroup 6-1 and the average value of correlation variation coefficients of the seven coefficients is 0.049. Relatively speaking, the compressive stress has a negligible influence on the influence patterns of other factors.

## 4. Conclusions

Influences of factors (temperature, shear strain rate, shear strain amplitude, and compressive stress) on the mechanical properties of HDR plate and coupling correlation are studied by tests and data analysis in this study. The conclusions are as follows:

(1) Temperature generally affects all coefficients greatly and is almost decisive for the initial modulus and the characteristic stress. Shear strain amplitude also has a great influence on each coefficient, while the shear strain rate on each coefficient is relatively small, and the influence of compressive stress is almost negligible.

(2) Shear strain amplitude has the greatest influence on the influence pattern of other factors. Temperature generally has the second greatest influence on the influence pattern of other factors. The influence of shear strain rate on the influence pattern of other factors is slight. Compressive stress has a negligible influence on the influence pattern of other factors.

Based on the above, future research can focus on three factors: temperature, shear strain amplitude, and shear strain rate, and the influence of compressive stress can be ignored.

## Figures and Tables

**Table 1:** Test cases.

Group 1: Correlation test between temperature and shear strain rate (15 cases)			
Temperature	Shear strain rate	Shear strain amplitude	Compression stress
−40 °C, −20 °C, 0 °C, 20 °C, 40 °C	0.4/s, 1.6/s, 6.4/s	150%	10 MPa
Group 2: Correlation test between temperature and shear strain amplitude (15 cases)			
Temperature	Shear strain rate	Shear strain amplitude	Compression stress
−40 °C, −20 °C, 0 °C, 20 °C, 40 °C	0.4/s	100%, 200%, 300%	12 MPa
Group 3: Correlation test between temperature and compressive stress (15 cases)			
Temperature	Shear strain rate	Shear strain amplitude	Compression stress
−40 °C, −20 °C, 0 °C, 20 °C, 40 °C	1.6/s	100%	6 MPa, 8 MPa, 12 MPa
Group 4: Correlation test between shear strain rate and shear strain amplitude (9 cases)			
Temperature	Shear strain rate	Shear strain amplitude	Compression stress
20 °C	0.4/s, 1.6/s, 6.4/s	50%, 150%, 250%	6 MPa
Group 5: Correlation test between shear strain rate and compressive stress (9 cases)			
Temperature	Shear strain rate	Shear strain amplitude	Compression stress
20 °C	0.4/s, 1.6/s, 6.4/s	200%	6 MPa, 8 MPa, 12 MPa
Group 6: Correlation test between shear strain amplitude and compressive stress (9 cases)			
Temperature	Shear strain rate	Shear strain amplitude	Compression stress
20 °C	0.4/s	50%, 150%, 250%	6 MPa, 8 MPa, 12 MPa

**Table 2:** Coefficients *C_i_* of Group *j*.

	X	x_1_	…	x_k_	…	x_m_	Variation Coefficient of *X*	Average Value of Variation Coefficients of *X*
Y								
y_1_		$C_{i,x_{1},y_{1}}$	…	$C_{i,x_{k},y_{1}}$	…	$C_{i,x_{m},y_{1}}$	$Cv_{i,X,y_{1}}$	$\mu_{Cv_{i},X(Y)}$
…		…	…	…	…	…	…	
y_l_		$C_{i,x_{1},y_{l}}$	…	$C_{i,x_{k},y_{l}}$	…	$C_{i,x_{m},y_{l}}$	$Cv_{i,X,y_{l}}$	
…		…	…	…	…	…	…	
y_n_		$C_{i,x_{1},y_{n}}$	…	$C_{i,x_{k},y_{n}}$	…	$C_{i,x_{m},y_{n}}$	$Cv_{i,X,y_{n}}$	
Variation coefficient of *Y*		$Cv_{i,x_{1},Y}$	…	$Cv_{i,x_{k},Y}$	…	$Cv_{i,x_{m},Y}$	——	——
Average value of variation coefficients of *Y*		$\mu_{Cv_{i},Y(X)}$					——	——

**Table 3:** Average values of variation coefficients for all the coefficients.

Subgroup	*X*	*Y*	*C*_1_	*C*_2_	*C*_3_	*C*_4_	*C*_5_	*C*_6_	*C*_7_
1-1	Temperature	Shear strain rate	0.210	0.477	0.482	0.478	0.419	0.477	0.472
1-2	Shear strain rate	Temperature	0.154	0.165	0.190	0.165	0.106	0.200	0.205
2-1	Temperature	Shear strain amplitude	0.122	0.369	0.379	0.369	0.348	0.374	0.371
2-2	Shear strain amplitude	Temperature	0.081	0.738	0.699	0.674	0.139	0.564	0.555
3-1	Temperature	Compression stress	0.283	0.584	0.587	0.584	0.541	0.578	0.570
3-2	Compression stress	Temperature	0.032	0.077	0.069	0.077	0.068	0.125	0.126
4-1	Shear strain rate	Shear strain amplitude	0.106	0.312	0.391	0.310	0.149	0.376	0.375
4-2	Shear strain amplitude	Shear strain rate	0.205	0.776	0.736	0.729	0.173	0.401	0.389
5-1	Shear strain rate	Compression stress	0.082	0.097	0.089	0.097	0.133	0.203	0.201
5-2	Compression stress	Shear strain rate	0.050	0.092	0.089	0.092	0.081	0.168	0.170
6-1	Shear strain amplitude	Compression stress	0.201	0.738	0.699	0.687	0.299	0.475	0.456
6-2	Compression stress	Shear strain amplitude	0.050	0.068	0.065	0.069	0.122	0.085	0.084

**Table 4:** Correlation variation coefficients of factor *Y* to *X*.

Subgroup	*X*	*Y*	*C*_1_	*C*_2_	*C*_3_	*C*_4_	*C*_5_	*C*_6_	*C*_7_
1-1	Temperature	Shear strain rate	0.062	0.032	0.027	0.032	0.018	0.061	0.063
1-2	Shear strain rate	Temperature	0.157	0.273	0.285	0.273	0.235	0.170	0.165
2-1	Temperature	Shear strain amplitude	0.377	0.199	0.198	0.199	0.121	0.238	0.242
2-2	Shear strain amplitude	Temperature	0.551	0.183	0.200	0.202	0.184	0.253	0.261
3-1	Temperature	Compression stress	0.044	0.052	0.042	0.052	0.075	0.073	0.075
3-2	Compression stress	Temperature	0.371	0.273	0.296	0.274	0.514	0.203	0.202
4-1	Shear strain rate	Shear strain amplitude	0.075	1.205	1.179	1.204	0.364	0.387	0.357
4-2	Shear strain amplitude	Shear strain rate	0.075	0.074	0.105	0.093	0.484	0.467	0.437
5-1	Shear strain rate	Compression stress	0.038	0.162	0.178	0.161	0.238	0.028	0.033
5-2	Compression stress	Shear strain rate	0.110	0.163	0.186	0.163	0.361	0.062	0.069
6-1	Shear strain amplitude	Compression stress	0.011	0.020	0.024	0.019	0.146	0.062	0.064
6-2	Compression stress	Shear strain amplitude	0.215	0.105	0.091	0.111	0.457	0.626	0.606

## Data Availability

The original contributions presented in this study are included in the article. Further inquiries can be directed to the corresponding author.

## Appendix A

**Table A1:** Coefficients *C_i_* at different temperatures and shear strain rates.

Temperature (°C)	Shear Strain Rate (/s)	*C*_1_	*C*_2_	*C*_3_	*C*_4_	*C*_5_	*C*_6_	*C*_7_
40	0.4	0.583	0.279	−0.957	0.923	0.219	2.228	−2.135
	1.6	0.655	0.332	−1.199	1.100	0.161	2.030	−1.955
	6.4	0.831	0.450	−1.741	1.490	0.164	1.417	−1.345
20	0.4	0.669	0.410	−1.444	1.357	0.274	3.508	−3.346
	1.6	0.821	0.512	−1.885	1.693	0.237	2.958	−2.806
	6.4	0.966	0.607	−2.321	2.009	0.208	2.439	−2.291
0	0.4	0.813	0.654	−2.369	2.164	0.351	5.290	−4.990
	1.6	1.003	0.936	−3.472	3.099	0.361	5.546	−5.231
	6.4	1.178	1.001	−3.809	3.314	0.302	3.436	−3.187
−20	0.4	0.953	1.000	−3.637	3.311	0.528	7.905	−7.426
	1.6	1.083	1.213	−4.545	4.014	0.466	7.305	−6.880
	6.4	1.286	1.060	−3.995	3.508	0.405	4.294	−3.991
−40	0.4	0.998	1.148	−4.168	3.801	0.668	10.780	−10.157
	1.6	1.207	1.389	−5.117	4.596	0.585	8.190	−7.679
	6.4	1.605	1.877	−7.111	6.215	0.547	6.124	−5.651

**Table A2:** Coefficients *C_i_* at different temperatures and shear strain amplitudes.

Temperature (°C)	Shear Strain Amplitude (%)	*C*_1_	*C*_2_	*C*_3_	*C*_4_	*C*_5_	*C*_6_	*C*_7_
40	100	0.560	0.188	−0.671	0.652	0.199	1.803	−1.773
	200	0.716	0.672	−2.280	2.104	0.251	5.742	−5.474
	300	0.796	2.152	−6.831	5.945	0.309	11.882	−11.071
20	100	0.642	0.284	−1.027	0.982	0.243	2.988	−2.892
	200	0.813	1.046	−3.628	3.277	0.312	9.483	−9.008
	300	0.837	2.482	−7.947	6.858	0.355	14.108	−13.165
0	100	0.756	0.417	−1.526	1.443	0.355	4.537	−4.357
	200	0.927	1.452	−5.075	4.548	0.445	11.143	−10.444
	300	0.883	2.700	−8.594	7.462	0.460	14.442	−13.359
−20	100	0.821	0.445	−1.644	1.541	0.418	4.708	−4.496
	200	0.930	1.475	−5.118	4.620	0.513	12.241	−11.523
	300	0.881	2.596	−8.223	7.173	0.548	17.254	−16.010
−40	100	0.953	0.670	−2.480	2.317	0.610	6.763	−6.414
	200	0.979	1.010	−3.478	3.165	0.587	7.670	−7.139
	300	0.988	5.882	−19.060	16.253	0.800	35.671	−33.311

**Table A3:** Coefficients *C_i_* at different temperatures and compressive stresses.

Temperature (°C)	Compressive Stress (MPa)	*C*_1_	*C*_2_	*C*_3_	*C*_4_	*C*_5_	*C*_6_	*C*_7_
40	6	0.555	0.168	−0.649	0.582	0.147	1.064	−1.043
	8	0.536	0.160	−0.601	0.554	0.131	1.122	−1.103
	12	0.590	0.196	−0.720	0.678	0.144	1.494	−1.464
20	6	0.684	0.255	−0.982	0.883	0.195	1.740	−1.677
	8	0.710	0.292	−1.121	1.010	0.204	1.984	−1.918
	12	0.723	0.312	−1.167	1.081	0.207	2.327	−2.247
0	6	0.905	0.516	−1.990	1.785	0.315	3.162	−3.005
	8	0.908	0.484	−1.860	1.673	0.350	3.137	−2.976
	12	0.937	0.595	−2.253	2.059	0.344	4.149	−3.955
−20	6	1.049	0.783	−3.009	2.709	0.442	4.912	−4.654
	8	1.115	0.945	−3.633	3.270	0.533	6.855	−6.499
	12	1.185	0.970	−3.699	3.357	0.583	6.358	−6.009
−40	6	1.207	1.036	−3.957	3.584	0.597	6.405	−6.042
	8	1.302	1.122	−4.258	3.881	0.764	7.639	−7.204
	12	1.294	1.048	−4.070	3.627	0.739	7.616	−7.185

**Table A4:** Coefficients *C_i_* at different shear strain rates and shear strain amplitudes.

Shear Strain Rate (/s)	Shear Strain Amplitude (%)	*C*_1_	*C*_2_	*C*_3_	*C*_4_	*C*_5_	*C*_6_	*C*_7_
0.4	50	0.490	0.097	−0.345	0.344	0.194	1.141	−1.139
	150	0.754	0.511	−1.831	1.692	0.292	3.517	−3.311
	250	0.833	1.287	−4.170	3.792	0.367	5.498	−4.977
1.6	50	0.591	0.159	−0.673	0.564	0.160	0.699	−0.696
	150	0.853	0.582	−2.152	1.927	0.239	2.410	−2.255
	250	0.932	1.336	−4.414	3.934	0.276	3.690	−3.338
6.4	50	0.621	−0.009	0.102	−0.030	0.189	2.768	−2.657
	150	1.002	0.619	−2.380	2.050	0.204	1.989	−1.834
	250	1.069	1.314	−4.395	3.871	0.217	2.591	−2.303

**Table A5:** Coefficients *C_i_* at different shear strain rates and compressive stresses.

Shear Strain Rate (/s)	Compressive Stress (MPa)	*C*_1_	*C*_2_	*C*_3_	*C*_4_	*C*_5_	*C*_6_	*C*_7_
0.4	6	0.793	0.741	−2.506	2.320	0.320	4.524	−4.218
	8	0.810	0.711	−2.410	2.229	0.324	4.413	−4.117
	12	0.890	0.863	−2.906	2.702	0.413	6.355	−5.961
1.6	6	0.890	0.803	−2.816	2.515	0.254	3.216	−2.995
	8	0.918	0.790	−2.755	2.475	0.260	2.928	−2.711
	12	0.985	0.936	−3.239	2.932	0.294	4.348	−4.057
6.4	6	0.959	0.604	−2.156	1.892	0.248	2.893	−2.732
	8	0.995	0.624	−2.213	1.955	0.264	2.990	−2.820
	12	1.094	0.773	−2.747	2.420	0.284	4.008	−3.783

**Table A6:** Coefficients *C_i_* at different shear strain amplitudes and compressive stresses.

Shear Strain Amplitude (%)	Compressive Stress (MPa)	*C*_1_	*C*_2_	*C*_3_	*C*_4_	*C*_5_	*C*_6_	*C*_7_
50	6	0.496	0.103	−0.380	0.365	0.190	1.047	−1.047
	8	0.500	0.105	−0.380	0.373	0.207	1.333	−1.323
	12	0.558	0.122	−0.439	0.434	0.242	1.448	−1.427
150	6	0.767	0.549	−1.970	1.817	0.313	4.221	−3.992
	8	0.765	0.487	−1.717	1.612	0.309	3.638	−3.438
	12	0.825	0.572	−2.007	1.895	0.357	4.810	−4.544
250	6	0.803	1.168	−3.758	3.439	0.367	5.345	−4.890
	8	0.830	1.186	−3.819	3.494	0.424	5.215	−4.787
	12	0.924	1.333	−4.257	3.928	0.582	5.245	−4.749
